# The intraocular implant and visual rehabilitation improve the quality of life of elderly patients with geographic atrophy secondary to age-related macular degeneration

**DOI:** 10.1007/s00417-022-05803-6

**Published:** 2022-08-19

**Authors:** Jana Nekolova, Jan Kremlacek, Jiri Lukavsky, Radovan Sikl, Martin Sin, Jana Langrova, Jana Szanyi, Nada Jiraskova

**Affiliations:** 1 Department of Ophthalmology, University Hospital Hradec Kralove and Faculty of Medicine, Charles University, Hradec Kralove, Czech Republic; 2 Department of Biophysics, Faculty of Medicine, Charles University, Simkova 870, 50003 Hradec Kralove, Czech Republic; 3 Department of Pathological Physiology, Faculty of Medicine, Charles University, Simkova 870, Hradec Kralove, 50003 Czech Republic; 4 Institute of Psychology, Czech Academy of Sciences, Brno, Czech Republic; 5 Military University Hospital Prague, Department of Ophthalmology 1st Faculty of Medicine of Charles University and Military University Hospital Prague, Prague, Czech Republic

**Keywords:** Age-related macular degeneration, Near vision, Quality of life, Scharioth macula lens

## Abstract

**Introduction:**

The objective of this prospective study was to evaluate the effects of intraocular macular lens implantation and visual rehabilitation on the quality of life of patients with geographic atrophy (GA) secondary to age-related macular degeneration (AMD).

**Methods:**

Patients with bilaterally decreased near vision (not better than 0.3 logMAR with the best correction), pseudophakia, were included in the project. The Scharioth macula lens (SML) was implanted into the patients’ better-seeing eye. Intensive visual rehabilitation of the ability to perform nearby activities was performed for 20 consecutive postoperative days. All subjects were examined before and after SML implantation ophthalmologically. The National Eye Institute 25-Item Visual Function Questionnaire (NEI VFQ-25) was administered before and 6 months after surgery.

**Results:**

Twenty eligible patients with mean age 81 years (63 to 92 years) were included in the project: 7 males and 13 females. Nineteen of them completed the 6-month follow-up. Near uncorrected visual acuity was 1.321 ± 0.208 logMAR before SML implantation and improved to 0.547 ± 0.210 logMAR after 6 months (*dz* =  − 2.846, *p* < 0.001, BF_10_ = 3.29E + 07). In the composite score of the NEI VFQ-25, there was an improvement in the general score and the specific domains related to the implantation. Participants reported fewer difficulties in performing near activities (*dz* = 0.91, *p* = 0.001, BF_10_ = 39.718) and upturns in mental health symptoms related to vision (*dz* = 0.62, *p* = .014, BF_10_ = 3.937).

**Conclusion:**

SML implantation, followed by appropriate rehabilitation, improved near vision and increased the quality of life of visually handicapped patients with AMD in our project.

**Supplementary Information:**

The online version contains supplementary material available at . 10.1007/s00417-022-05803-6.


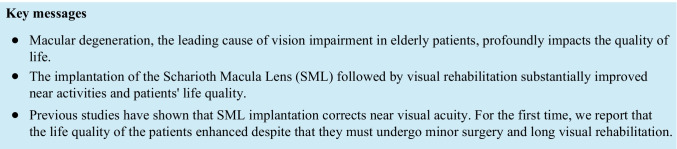


## Introduction

A leading cause of visual impairment in elderly patients is age-related macular degeneration (AMD) [1]. AMD management is complicated and does not lead to the complete recovery of visual function. Despite many new therapeutic methods, the disease still leads to a progressive loss of central vision and impedes the ability to perform activities such as reading or recognizing details [2, 3]. Macular degeneration has a deeply negative impact on the quality of life of elderly patients and disables their capacity to perform their daily activities [4, 5].

There are several types of vision-improving magnifying devices to help patients with vision impairment due to maculopathy. Extraocular devices include binocular spectacles, telescopes for distant vision improvement, and several types of hand-held magnifiers to help patients read again. However, optical aids for low vision have many limitations, such as constricted visual fields, even though the device is relatively easy to manipulate effectively [6]. Understanding the principles and controls of currently available optoelectronic external aids is demanding for elderly patients [7, 8].

Technological advancement in the field of intraocular implants for low vision creates new hope for patients with AMD [9]. Among other implants, the Scharioth macula lens (SML) is a bifocal Add-On intraocular lens (IOL) with a specifically designed central optic area providing a high addition power of + 10.0 D. It is intended for monocular implantation in the better-seeing pseudophakic eye in patients with decreased near vision due to maculopathy [10]. The SML provides a hypercorrection that boost near visual acuity as several studies proved [9–12]. This magnification is ready to use without any preparation, follows the gaze, and does not occupy hands. However, the implantation brings a substantial stress and afraid of a surgery, the resulting reading distance is unusually short, the implant is not adjustable, it is not easily removable from the eye, and the SML subtly changes distant and peripheral vision [9]. Only a complex assessment can answer the question of whether implantation of the SML is beneficial for a patient with geographic atrophy [11–14].

Several visual tests and measurements fail to completely describe the emotions of a patient with decreased vision and the extent to which they are handicapped by the condition. Vision function and vision-related quality of life questionnaires are used to evaluate the problems with vision and the emotions that the patients experience regarding their vision condition [15]. It can be useful to describe the efficacy of methods that help people with central vision loss [4]. However, it may not completely reflect the distress resulting only from visual disability, but the patient’s general condition also plays an important role. Well-being is an important socioeconomic outcome that reflects how people perceive their life from their own perspectives. Patients with visual impairments have lower satisfaction with life than those without visual impairments [16]. It is crucial to focus attention on the psychological impacts of the disease on the patient rather than focusing solely on ocular pathologies.

In the present study, we evaluated how implantation of an intraocular macular lens followed by visual rehabilitation influences the quality of life of patients with decreased near vision due to geographic atrophy secondary to AMD. Of the currently available literature, no studies have evaluated this topic.

## Methods

Patients aged 63 years or older with decreased near vision due to GA were included in this 3-year-long prospective study. Written informed consent was obtained from all participants following an explanation of all procedures involved. The protocol respected the tenets of the Declaration of Helsinki. The study was approved by the Ethical Committee of the University Hospital in Hradec Kralove, Czech Republic.

All participants met the inclusion criteria for implantation of the Scharioth macula Lens. According to the recommendations of the manufacturer (Medicontur International, Geneva, Switzerland) [17], the SML is suitable for motivated patients with dry AMD and near vision difficulties who are pseudophakic and who show sufficient near vision improvement on near vision tests performed with standard near correction at 40 cm and with + 6.0 D at 15 cm. DBCVA should range from logMAR + 0.5 to + 1.0. Exclusion criteria are active neovascular AMD/maculopathy, active iris neovascularization, shallow pseudophakic ACD (< 2.8 mm; from endothel), narrow angle, uveitis, pupillary abnormalities (photopic pupil less than 2.5 mm), corneal diseases involving central cornea, non-central scotomas, and other severe ocular diseases.

The intraocular implant, the SML, was implanted into the better-seeing eye of the patients, based on the manufacturer’s recommendations.

All participants underwent cataract surgery at least 3 months before SML implantation.

### Surgical procedure and ophthalmological examination

All subjects were examined before and after SML implantation. Near visual acuity (NVA) was measured using the Jaeger chart; the paragraphs of text size increased from 0.37 mm (J1) to 50.1 mm (J24). The smallest print that the patient could read determined his or her NVA. The near logMAR VA was counted as the angle of 1/5 of the height of the letter “a” in the Jaeger chart. Reading from a distance of 175 mm, J15 text size corresponds to logMAR 1.3, J10 to 0.9, J5 to 0.6, and J1 to logMAR 0.4.

The distant vision was tested using electronic optotypes from 6 m and was reported in logMAR. Optical coherence tomography (OCT) was used to obtain detailed images from within the retina and to measure central retinal thickness, and it was performed on a Cirrus HD-OCT Model 4000 (Carl Zeiss, Germany). Intraocular pressure was measured with air-puff tonometry. The anterior segment and then the posterior segment of eyes in artificially dilated pupils were examined, and digital photographs together with fundus autofluorescence were obtained. Corrected NVA was tested preoperatively with + 3.0 D from a distance of 350 mm and with + 6.0 D from a distance of 150–175 mm and from the same distance without correction postoperatively.

Michelson contrast sensitivity was measured with Landolt C with an outer diameter of 480′ on a computer monitor with a resolution of 1024 × 768 pixels. The image observed from a distance of 0.6 m was administered by the Freiburg Visual Acuity Test with 4 choices in 24 trials [18]. Non-study eyes were also examined during the study period.

Computer testing of the visual field with a Zeiss Humphrey field analyzer, Model 745i, Dublin, CA, USA perimeter, was performed before SML implantation to exclude patients other than central scotomas.

Selection of participants, implantation of the SML, clinical care, and ophthalmological examination were conducted at the Department of Ophthalmology, University Hospital in Hradec Kralove, Czech Republic. All implantations were performed by one surgeon (N.J.). After pupil dilation and topical anesthesia, an incision of 2.2 mm was made. The anterior chamber was filled with an ophthalmic viscosurgical device (OVD), and the SML was implanted using a cartridge into the sulcus in front of the artificial lens. After implantation, proper positioning of the haptics in the ciliary sulcus and IOL centration were adjusted. Finally, the OVD was removed, and the incisions were hydrated. All patients underwent standard postoperative treatment with topical antibiotics for 1 week and topical steroids for 1 month.

### Visual rehabilitation

Intensive visual rehabilitation under the supervision of a professional therapist was performed every day for 20 consecutive days from the second day after surgery. Appropriate and repetitive training was used to improve visual skills of localization, fixation, scanning, and tracing of an object in the near visual scene. Patients were trained to use the eye with SML for reading and for performing near activities. Sharp vision was achieved at a very near distance of 10–15 cm with good illumination of the text. Patients were asked to read text from the largest to the smallest type and to recognize different shapes. A proper orientation in a text was also practiced. Glasses or external magnifiers were not required for the reading.

### Quality of life

Two vision-specific quality of life questionnaires were used to assess the impact of the intervention on the vision-related quality of life (VRQoL) of the participants. The National Eye Institute 25-Item Visual Function Questionnaire (NEI VFQ-25) [19] was administered twice. The questionnaire was first administered before SML implantation and then again 6 months after surgery. The questionnaire was read aloud to the participants by a medical professional not involved in previous assessments or rehabilitation, who also noted their responses. The NEI VFQ-25 consists of 12 short subscales ranging from 0 to 100 (higher values correspond to a better quality of life). The composite score can be calculated as an average of 11 subscales (excluding the visually nonspecific general health subscale). Because the participants could not drive, we also excluded the driving subscale and calculated the composite score from the remaining ten subscales. We report the preintervention and postintervention scores and their differences. The psychometric validation of the NEI VFQ-25 questionnaire was done for the conditions of the Slovak Republic and was published in the Journal of Czech and Slovak Ophthalmology [20]. Slovakia is culturally and socially very close to the Czech Republic environment and speakers of both countries understand each other without a translation. So far, several studies using the NEI VFQ-25 in the Czechoslovak environment have been conducted [21–23]. The Czech version of the NEI VFQ-25 was created and validated according to the recommendation for linguistic validation of the questionnaires [24]. The initial translation from the original language to the Czech language was done by two independent translators (K.S., J.L.), the versions were compared, and the best interpretation was chosen. It was then independently back-translated to the English language by a native speaker (C.R.) and compared to the original. There were no discrepancies between back-translated and original English versions. We also compared the Czech and Slovak versions of NEI VFQ-25, and we did not find any variances.

The second questionnaire was administered once, 6 months after surgery. It was inspired by the Macular Disease Quality of Life (MacDQoL) [25] questionnaire but largely modified to fit the specifics of the situation.

First, based on the discussion with the vision rehabilitation therapists who were aware of the patients and their needs, some of the items of the questionnaire were changed. Specifically, 7 items of the original questionnaire were omitted because they were not considered informative, while 3 other items concerning patients’ reading, writing, and watching TV were added. The questionnaire we used thus consisted of 19 items instead of 23.

Second, the values on the 5-point scale were relabeled as much worse, slightly worse, the same, slightly better, and much better to evaluate the hypothesized postinterventional improvements.

The third and most important difference concerned the formulation of the question and hence the mental operations the patients were asked to perform. They were not simply reporting the current state. Instead, they compared it to the hypothetical situation in which they did not undergo implantation and directly evaluated the benefit of surgery and rehabilitation.

Fourth, unlike the authors of the MacDQoL questionnaire, we only asked the patients to assess the impact of the intervention on a particular life domain and not the importance they assign to that domain.

For all these modifications, the data collected from the questionnaire inspired by MacDQoL were not evaluated independently and are not directly comparable to other MacDQoL studies.

### Data analysis

The statistical analysis was performed in R [26] and JASP Computer software (Version 0.14.1), which was used only for Bayes factors in Wilcoxon tests. We report means and standard deviations for the selected vision parameters. The differences were evaluated using Wilcoxon paired tests, and we report mean differences and their SD, *p* values, and Bayes factors (BF_10_). Bayes factors allow researchers to report evidence for both alternative and null hypotheses; consequently, we can argue that visual function or aspects of quality of life did not change after implantation.^1^ In the case of NEI VFQ-25 measures, we analyzed the differences using paired *t* tests.

## Results

Twenty patients with near vision impairment due to GA resulting from AMD were included in the project, 7 males and 13 females. Nineteen of them completed the 6-month follow-up, and one patient died of pneumonia before the end of the study at age 89. There was no relation between the cause of death and the study method or regimen. The average age of participants at the time of implantation was 80.5 years (min. 63 years, max. 92 years). In the first year of the project (2018), four participants were recruited; in 2019, eight were recruited and in 2020, eight patients were included.

### Surgical procedure and ophthalmological examination

Table 1 shows the distance best corrected visual acuity (DBCVA), near uncorrected visual acuity (NUVA), and near visual acuity examined with correction + 3 D (NCVA3) and + 6.0 D (NCVA6). The mean and standard deviation of pre- and post-operative acuities and their differences are listed in logMAR units.

**Table 1:** Summary of the distance best corrected visual acuity (DBCVA, in logMAR units), the near uncorrected visual acuity (NUVA), the near corrected visual acuity with + 3 D (NCVA3) and + 6 D (NCVA6) in logMAR units, and the Michelson contrast sensitivity (Landolt contrast) in percents. The difference was calculated as the end of follow-up minus the preintervention value. NUVA at the end of follow-up was used for NCVA3 and NCVA6 differences. Effect sizes are expressed as Cohen *d*z; *p* values correspond to Wilcoxon paired tests; *N* number of patients, *M* mean value, *SD* standard deviation, *BF*_*10*_ Bayes factor

Vision measurement	*N*	Before intervention		After 6 months		Difference		*d*z	*p*	BF_10_
		*M*	SD	*M*	SD	*M*	SD			
DBCVA	19	0.662	0.228	0.737	0.166	0.074	0.191	0.388	0.033	1.84
NUVA	19	1.321	0.208	0.547	0.210	− 0.774	0.272	− 2.846	< 0.001	3.29E + 07
NCVA3	19	0.919	0.211			− 0.372	0.181	− 2.052	< 0.001	289,724.34
NCVA6	19	0.557	0.133			− 0.010	0.170	− 0.060	0.593	0.25
Landolt contrast	19	4.469	5.157	5.445	5.125	0.976	2.677	0.365	0.087	1.26

Preoperatively, the DBCVA of all participants ranged from 0.39 to 1.30 logMAR. This was in accordance with the inclusion criteria of SML implantation. Near visual acuity corrected with conventional addition (+ 3.0 D for pseudophakic eyes with refractive purpose of zero diopters for distance) was equal to or worse than J7 and there was significant improvement of at least 3 sizes of Jaeger chart when corrected with + 6.0 D.

The SML implantation procedures and post-implantation recovery were without any complications or adverse events in all subjects. During the 6-month period, two participants were treated for adenoviral keratoconjunctivitis with no relationship to the study procedure, and they fully recovered. One of the patients underwent Nd:YAG capsulotomy during the rehabilitation period.

Postoperatively, there was a decrease in DBCVA (*p* = 0.033, see Table 1), but the change was relatively small. The DBCVA was worse in 13 patients; in 5 patients, the DBCVA improved slightly, and in 1 participant, it remained stable. We observed improvements in near visual acuity; the changes were substantial — the postimplantation state was better than both the preoperative uncorrected acuity and the acuity with + 3.0 D correction. Our measurements provided weak to moderate evidence for the idea that postimplantation NVA was analogous to the preimplantation + 6.0 D correction (BF_10_ = 0.24). In 8 patients, postoperative NVA was equal to + 6.0 D corrected NVA; in 4 of them, it was better, and 7 participants read bigger letters of the Jaeger test compared to preoperative + 6.0 D corrected results (Table 1).

There was no significant change in contrast sensitivity measured by Landolt rings in our series before and 6 months after SML implantation (*p* = 0.087, see Table 1). The function of the non-study eyes without SML remained also unchanged in most cases; contrast sensitivity of the fellow eye was 16.01% (SD 30.07) in the beginning of the study and after 6 months it was 16.69% (SD 29.93); *p* value of the Wilcoxon paired test was 0.320.

Mean central retinal thickness (CRT) measured with OCT was 191 μm (min. 107, max. 267 μm) preoperatively, and in all cases, there was localized sharply demarcated atrophy of outer retinal tissue, retinal pigment epithelium, and choriocapillaris, in some cases together with RPE deformation or thickening by drusen. There was no edema or abnormal neovascular tissue. AMD of all study eyes was graded as advanced geographic atrophy according to Ferris et al. [27]. Study eye was the better-seeing eye of the patient. All participants had bilateral AMD. there were 18 patients with advanced GA in the contralateral eye and 2 patients with advanced neovascular AMD of the non-study eye. The fovea was involved in all the study eyes. We used the fundus image to estimate the GA area, measured the macular lesion’s horizontal and vertical diameter, and then calculated the elliptic area. The mean and range of the vertical diameter of the GA lesion was 2918.8 μm (min. 993, max.5957 μm), the horizontal diameter subtended 2985 μm (min. 1266, max. 5842 μm) and the estimated elliptical area was 8.4 mm2 (min. 1.0, max. 27.3 mm2). All patients met the indication criteria for SML implantation. They could not read the newspaper text with the best glass correction and refused to use external magnifiers because of stigmatization.

At the end of follow-up, the mean CRT was 181 μm (min. 99, max. 284 μm). During the study period of 6 months, there was no significant progression of AMD or enlargement of GA. There was no shift to neovascular AMD during a 6-month follow-up period. Mean central retinal thickness of fellow eyes was 212 μm (min. 33, max. 539 μm) and did not significantly change during the follow-up period.

Intraocular pressure remained unchanged comparing preoperative and postoperative data; the mean IOP was 14.5 mmHg (min. 10, max. 21 mmHg) before and 14 mmHg (min. 9, max. 22 mmHg) after SML implantation. There was no severe elevation of IOP caused by the surgery.

### Vision-related quality of life

The VRQoL reports are summarized in Table 2. In the composite score of the NEI VFQ-25, we observed a general improvement (*dz* = 0.65, *p* = 0.011, BF_10_ = 4.734), which was also supported by a significant positive change in the general vision subscale (*dz* = 0.75, *p* = 0.004, BF_10_ = 10.942). Participants reported improvements in the specific domains related to the implantation. They reported fewer difficulties in performing near activities (*dz* = 0.91, *p* = 0.001, BF_10_ = 39.718) and improvements in mental health symptoms related to vision (*dz* = 0.62, *p* = 0.014, BF_10_ = 3.937). We found weak evidence for feeling less dependent on others (*dz* = 0.50, *p* = 0.042, BF_10_ = 1.612). The remaining domains were not affected, and the Bayes factors indicate anecdotal evidence for no change (BF_10_ in the 0.2 to 0.33 range). The near activities domain change related to the intervention was negatively related to the size of GA (*r* =  − 0.54,* p* = 0.017).

**Table 2:** NEI VFQ-25 scores before intervention and 6 months later. Effect sizes are expressed as Cohen *dz*, and *p* values correspond to paired *t* tests. *N* number of patients, *M* mean value, *SD* standard deviation, *BF*_*10*_ Bayes factor

NEI VFQ-25 scale	*N*	Before intervention		After 6 months		Difference		*d*z	*p*	BF_10_
		*M*	SD	*M*	SD	*M*	SD			
General health	19	40.8	12.4	39.5	12.7	− 1.3	15.5	− 0.08	0.716	0.25
General vision	19	49.5	15.4	61.1	14.1	11.6	15.4	0.75	0.004	10.94
Ocular pain	19	90.8	14.3	91.4	13.2	0.7	17.9	0.04	0.875	0.24
Near activities	19	37.5	22.9	57.2	24.2	19.7	21.7	0.91	0.001	39.72
Distance activities	18	52.1	26.0	50.2	24.2	− 1.9	20.4	− 0.09	0.705	0.26
Vision-specific social functioning	18	70.8	21.4	81.2	22.8	10.4	23.6	0.44	0.078	1.02
Vision-specific mental health	19	42.4	15.5	54.3	19.0	11.8	19.0	0.62	0.014	3.94
Vision-specific role difficulties	19	46.7	24.2	48.7	19.9	2.0	25.4	0.08	0.739	0.25
Vision-specific dependency	19	50.9	21.9	62.7	24.4	11.8	23.6	0.50	0.042	1.61
Color vision	19	88.2	19.3	92.1	20.5	3.9	22.5	0.18	0.454	0.31
Peripheral vision	18	81.9	16.7	86.1	26.0	4.2	24.6	0.17	0.483	0.31
Composite score	19	60.8	10.7	68.3	13.1	7.5	11.6	0.65	0.011	4.73

When asked to directly compare the impact of the implantation on quality of life, the responses were in agreement with the NEI VFQ-25 results. People most often reported improvements in reading and household tasks. The other common responses included improvements in self-confidence, physical well-being, independence, and enjoyment of nature. The self-reported evaluation of the postimplantation state to the pre-operated state is shown in Table 3..

**Table 3:** The self-reported evaluation of the postimplantation state to the nonoperated state is sorted in ascending order (most positive assessments on top). *TV* television, *NA* not applicable

Item	Much worse	A little worse	Same	A little better	Much better	NA
Reading	1	2	2	7	7	
Handle household tasks		1	7	9	2	
Self-confidence			10	7	2	
Feel physically			11	5	3	
Do things independently			11	7	1	
Enjoy nature			11	5	3	
Enjoy life		1	9	6	2	1
Time required for activities			12	5	2	
Can watch TV		1	3	8		7
Physical appearance and care			12	6		1
Family and relationships			14	3	2	
Move outside		1	12	5	1	
Hobbies and interests		3	9	3	2	2
Enjoy meals and drinks			17	1	1	
People reactions to me			17	1		1
Lose things		1	16	2		
Writing	1	3	9	3	2	1
Social life and new contacts		4	12	3		
Experience of shopping	1	3	13		2	

## Discussion

Age-related macular degeneration harms the quality of life and the patients’ emotional status [28–30]. According to longitudinal studies evaluating the adaptation to AMD, there was a decrease in mood, life satisfaction, social functioning, and ability to perform activities of daily living and an increase in stress, depression, and requiring help from another person over a 5-year period [31, 32]. Depression in AMD has been reported to be strongly correlated with increasing VA loss [33].

One of the primary ways to compensate for impaired visual acuity is to magnify the object of interest. Among various low-vision implants [34], we evaluated the effect of the Scharioth macula lens on VRQoL as it should not suffer from a visual field restriction, diplopia, contrast, and brightness reduction. A clear fundoscopy after implantation is also an important advantage for follow-up examinations.

For many patients, the main drawback of the SML is the surgical procedure. Current knowledge shows that SML in a group of fifty patients did not cause intraoperative complications and had to be explanted only in one patient because of glare/halos [14]. Even more demanding cataract surgery does not increase the risk of pre-existing GA progression [35]. Equally, satisfactory visual outcomes and controlled disease activity were found in patients with wet AMD after cataract surgery [36]. There was no case of rapid worsening of GA, no change to neovascular AMD, and no need for anti-VEGF application after SML implantation in our study. According to our experience, the SML implantation is technically easier than cataract surgery and with minor trauma to the eye. There was no surgery complication or an explantation in our cohort of 20 patients. Preoperatively, reading with + 6.0 sphere diopters is believed to be equal to uncorrected NVA postoperatively [10, 37]. Such procedure is part of the inclusion criteria, and it helps patients to imagine the postoperative result and decreases patients’ anxiety. Our measurements provided weak to moderate evidence for this idea.

Along with the VRQoL, we also assessed changes in near and distant visual acuity and contrast sensitivity. Similarly, to outcomes of a European multicenter clinical trial [37] and other studies [10, 12, 13], we found a strong improvement in near vision. The benefit was optimal for a reading distance of 15 cm with adequate light.

Six months after SML implantation, we observed a mild decrease in distance visual acuity, although patients rated their distance activities equally. The time course of the visual acuity drop showed a slow trend suggesting an adaptive process of building monovision due to intensive use of the implanted eye for near activities [11]. We trained our patients to find their preferred retinal locus for near activities in the + 10 D magnifying area of the SML. This may interfere with their distant vision [38, 39] especially for a narrow pupil (we instructed patients to use sunglasses in direct sunlight for distant activities). Other studies of the SML did not report a significant change in distant visual acuity, likely because their effect was small [11].

Contrast sensitivity is another crucial factor affecting VRQoL in bilateral advanced age-related macular degeneration [40, 41]. In patients with AMD, contrast sensitivity decreases and is associated with a lower mean composite score in the NEI VFQ-25 questionnaire with a poor score in subcategories: near vision, distance vision, and dependency [42]. There was no significant change in contrast sensitivity with SML compared to preoperative data in our group of patients.

An essential part of the intervention was visual rehabilitation, which is necessary to help low-vision patients utilize aids to perform daily activities [43]. We targeted the visual rehabilitation to near activities and reading, which was reported by patients as a highly valued domain for their VRQoL in study inclusion.

To estimate the effect of the SML implantation, we searched the literature on the impact of an intervention on VRQoL. Comparisons can only be made to studies that used a different optical correction because there is no study on SML. On 23 Jun 2022, PubMed returned 12 items for a search “geographic atrophy” AND “VFQ-25.” Only two of them were prospective studies and relevant for the comparison.

In a multicenter study, Hudson et al. followed patients with the Implantable Miniature Telescope (IMT) [44] — one of the available implants aimed at improving GA vision [9, 45, 46]. The IMT causes a 2.2–3.0 × magnification of the image on the retina, with a sharp vision from 1.5 to 10 m. To bring the sharp vision to reading distance, IMT was supplemented with external spectacle correction. IMT implantation is more challenging than SML and limits the field of view to a central 20°–24°.

VRQoL was measured before and 1 year after implantation using the NEI VFQ-25 in 206 patients (75.6 ± 7.3 years, BCVA of 1.20 ± 0.22 logMAR). They found improvements in near and distance visual acuity accompanied by an increase in VRQoL. For near activities, the score improved from an initial 25.5 ± 14.2 to 37.3 ± 18.8, corresponding to an effect size of 0.58. They also found a statistically significant effect of 0.31 for distant activities, general vision, social functioning, mental health, role difficulties, and dependency. The score for peripheral vision significantly dropped (effect size =  − 0.19). The total score increased significantly from 43.9 ± 13.3 to 50.3 ± 14.7 — effect size 0.46 [44].

Another prospective study of ten patients with GA (82.5 ± 6.2 years, BCVA of 1.7 ± 0.4 logMAR) evaluated the change in VRQoL after cataract surgery [47]. One year after surgery, the near-activity score increased statistically insignificantly from 31.5 ± 5.6 to 39.8 ± 19.4. The patients reported improvement in mental health and role difficulties. The total score increased significantly from 44.0 ± 7.1 to 56.9 ± 15.6.

Our study observed a substantial effect of 0.91 for near activities and 0.65 for the composite score. Such a strong effect was not detected in the studies mentioned above. The higher effect in our study might be caused by intensive visual rehabilitation, absence of peripheral vision loss, and shorter a half-year follow-up.

The present study is not the first to explore longitudinal trends in VRQoL in AMD patients. A recent study compared longitudinal VRQoL progression in patients with GA and neovascular AMD in the periods before and after the development of advanced disease and found that the natural progression of VRQoL differed in both groups [48]. Patients with GA experienced a similar decline in VRQoL before and after developing the advanced disease, while patients with neovascular AMD experienced a dramatic decline in VRQoL after the transition to advanced AMD. Künzel et al. evaluated longitudinal VRQoL progression in patients with GA. Patients showed a slight, but not significant, decline between baseline and follow-up visits in the composite score and near and distant vision subscores [49]. In comparison, in the present study, a significant positive change was found in 4 of the 12 NEI VFQ-25 subscales, including the domains related to the implantation, while the remaining domains were unaffected.

The extent of the visual impairment also limits the intervention outcomes. Patnaik et al. [50] reported lower NEI-VFQ 25 scores among AMD patients with GA compared to neovascular AMD. In our study, the area of the GA was closely related to the VRQoL improvement for near activities.

In conclusion, we found that the implantation of the SML followed by visual rehabilitation improves the quality of life in patients with GA secondary to AMD. The benefit of intervention was restricted to reading and household tasks and depended on the GA size.

## Limitations

The measurement of VRQoL is subjective and is influenced by many factors, including mental disposition, mood, expectations, relationships, functional status, degree of disability, ability to adapt to new situations, and many others. Quality of life is measured via questionnaires or other forms of patient-reported outcome measures (i.e., PROMs). Together with performance-based measures, it can show the relationship between the pathophysiology of ocular disease and patient-reported functioning [51–54]. There are different strategies for completing questionnaires, which may also influence the results of the VRQoL measurement. The questionnaires are managed by the patient or by another person, the patient’s relatives, or a professional. Some patients require help with reading and some with response marking. A stressful environment while managing questionnaires should be avoided, and patients should feel comfortable. The purpose is to eliminate the temporary discomfort of the patient. In our project, the questionnaires were read aloud to the participants by a medical professional who also noted their responses in a quiet room. They were not involved in previous assessments or rehabilitation.

According to the choice of questionnaires, we preferred one standardized, widely used (the NEI VFQ-25). The psychometric validation and linguistic validation of the NEI VFQ-25 for the Czech environment were done. Another questionnaire was inspired by the MacDQoL questionnaire, which was largely modified to fit the specifics of the situation and targeted near vision activities and was not validated. We are aware that more detailed or longer questionnaires can show more specific outcomes; however, there is a strong limitation in elderly patients because of the increased fatigue and decreased attention during prolonged completion of the questionnaire. Elderly patients also have difficulty understanding some questions and need more explanation from the other person, which can also influence the results. In our study, the person administrating the questionnaires explained all questions if necessary and assured their understanding.

We experienced the negative effects of COVID-19 quarantine and lack of social contact and cultural life of elderly individuals on patients’ mood and happiness during the end of our study. The last four participants of this project were impacted by COVID-19 quarantine and, in accordance with all protective recommendations, were examined during the lockdown period. They reported a decrease in social functioning.

In our study, we primarily investigated whether an intervention combining macular lens implantation and visual rehabilitation would improve the quality of life of recipients. Thus, we cannot answer what the effectiveness of rehabilitation or implantation of the SML per se is. This question we address in a forthcoming study in which the implantation will be compared to an external hypercorrection.

## Supplementary Information

Below is the link to the electronic supplementary material.

Supplementary file1 (PDF 39 KB)

Supplementary file2 (DOCX 24 KB)

Supplementary file3 (DOC 55 KB)

Supplementary file4 (DOC 53 KB)

## Data Availability

The dataset is obtainable from the corresponding author on reasonable request.

## Footnotes

1 The suggested interpretation thresholds [55]: > 100 extreme evidence for H_A_, 30–100 very strong evidence for H_A_, 10–30 strong evidence for H_A_, 3–10 moderate evidence for H_A_, 1–3 anecdotal evidence for H_A_, 1 no evidence, 1/3–1 anecdotal evidence for H_0_, 1/10–1/3 moderate evidence for H_0_, 1/30–1/10 strong evidence for H_0_, 1/100–1/30 very strong evidence for H_0_, < 1/100 extreme evidence for H_0_.
