# Supplementary material for: The intraocular implant and visual rehabilitation improve the quality of life of elderly patients with geographic atrophy secondary to age-related macular degeneration
Source: Graefes Arch Clin Exp Ophthalmol. 2022 Aug 19;261(1):263–72. doi: 10.1007/s00417-022-05803-6 (PMC9388354; doi:10.1007/s00417-022-05803-6)
Supplement: Supplementary file 2 — Supplementary file2 (DOCX 24 KB) [file 417_2022_5803_MOESM2_ESM.docx]

VFQ-25

Verze 2000 - varianta předčítaná administrátorem

# Instrukce pro administrátora

Před první administrací si přečtěte dotazník. Při administraci čtěte otázky a nabízené odpovědi. Nabízené odpovědi jsou v řadě otázek stejné, pokud pacient odpovídá spontánně, nemusíte je číst.

Text VELKÝM PÍSMEM nečtěte, jsou to poznámky pro vás. Podtržený text zdůrazňuje těžiště otázky – je to informace pro vás, co při čtení zdůraznit, nebo co hlavně říci, pokud bude třeba otázku pacientovi zopakovat.

# Instrukce pro pacienta

Budu vám číst různé výroky o problémech, které se týkají vašeho zraku nebo vašich pocitů, které týkají se vaší poruchy zraku. Po každé otázce vám přečtu seznam možných odpovědí. Prosím vyberte odpověď, která nejlépe popisuje vaši situaci.

Prosím, odpovězte na všechny otázky, tak jako byste měl/a vaše brýle či kontaktní čočky (pokud je nosíte).

Na každou otázku máte tolik času, kolik potřebujete. Všechny vaše odpovědi jsou důvěrné. Pokud nám tento dotazník má pomoci zlepšit poznatky o zrakových problémech, a jaký vliv mají na kvalitu vašeho života, je potřeba, aby vaše odpovědi byly co nejpřesnější. Připomínám, že pokud pro nějakou činnost nosíte brýle či kontaktní čočky, odpovídejte na otázky, jako byste je měl na sobě.

# Část 1 – celkové zdraví a zrak

1) Řekl/a byste, že váš zdravotní stav je celkově:

PŘEČTĚTE a zakroužkujte jednu odpověď Výborný 1

Velmi dobrý 2

Dobrý 3

Přiměřený 4

Špatný 5

2) Řekl/a byste, že váš zrak, když použijete obě oči (a brýle nebo čočky, pokud je nosíte), je v současnosti:

PŘEČTĚTE a zakroužkujte jednu odpověď Výborný 1

Dobrý 2

Přiměřený 3

Špatný 4

Velmi špatný 5

Jste úplně slepý/á 6

3) Jak často si děláte starosti o svůj zrak?

PŘEČTĚTE a zakroužkujte jednu odpověď Vůbec nikdy 1

Málokdy 2

Občas 3

Často 4

Neustále 5

4) Jak velké bolesti či nepohodlí cítíte uvnitř a v okolí vašich očí (např. pálení, svědění, bolest)? Řekl/a byste, že:

PŘEČTĚTE a zakroužkujte jednu odpověď Žádné 1

Mírné 2

Střední 3

Velké 4

Velmi velké 5

# Část 2 – obtíže v činnostech

Následující otázky se týkají obtíží, které (možná) máte během různých činností, při kterých máte své brýle nebo čočky, pokud je používáte.

5) Jak je pro vás obtížné číst běžný text v novinách? Řekl/a byste, že to je:

PŘEČTĚTE KATEGORIE, POKUD BUDE TŘEBA, a zakroužkujte jednu odpověď

Není to vůbec obtížné 1

Trochu obtížné 2

Středně obtížné 3

Extrémně obtížné 4

Přestal jsem to dělat kvůli svému zraku 5

Přestal jsem to dělat kvůli jiným důvodům nebo mne to nezajímá 6

6) Jak obtížné jsou pro vás práce nebo koníčky, které vyžadují dobře vidět nablízko (jako je vaření, šití, domácí opravy nebo používání nářadí)? Řekl/a byste, že to je:

PŘEČTĚTE KATEGORIE, POKUD BUDE TŘEBA, a zakroužkujte jednu odpověď

Není to vůbec obtížné 1

Trochu obtížné 2

Středně obtížné 3

Extrémně obtížné 4

Přestal jsem to dělat kvůli svému zraku 5

Přestal jsem to dělat kvůli jiným důvodům nebo mne to nezajímá 6

7) Kvůli vašemu zraku, jak je pro vás obtížné najít něco na zaplněné polici?

PŘEČTĚTE KATEGORIE POKUD BUDE TŘEBA a zakroužkujte jednu odpověď

Není to vůbec obtížné 1

Trochu obtížné 2

Středně obtížné 3

Extrémně obtížné 4

Přestal jsem to dělat kvůli svému zraku 5

Přestal jsem to dělat kvůli jiným důvodům nebo mne to nezajímá 6

8) Jak je pro vás obtížné číst dopravní značky nebo názvy obchodů?

PŘEČTĚTE KATEGORIE POKUD BUDE TŘEBA a zakroužkujte jednu odpověď

Není to vůbec obtížné 1

Trochu obtížné 2

Středně obtížné 3

Extrémně obtížné 4

Přestal jsem to dělat kvůli svému zraku 5

Přestal jsem to dělat kvůli jiným důvodům nebo mne to nezajímá 6

9) Kvůli vašemu zraku, jak je pro vás obtížné za šera nebo v noci scházet po schodech či překračovat obrubníky?

PŘEČTĚTE KATEGORIE POKUD BUDE TŘEBA a zakroužkujte jednu odpověď

Není to vůbec obtížné 1

Trochu obtížné 2

Středně obtížné 3

Extrémně obtížné 4

Přestal jsem to dělat kvůli svému zraku 5

Přestal jsem to dělat kvůli jiným důvodům nebo mne to nezajímá 6

10) Kvůli vašemu zraku, jak je pro vás obtížné všimnout si předmětů po stranách, když je míjíte?

PŘEČTĚTE KATEGORIE POKUD BUDE TŘEBA a zakroužkujte jednu odpověď

Není to vůbec obtížné 1

Trochu obtížné 2

Středně obtížné 3

Extrémně obtížné 4

Přestal jsem to dělat kvůli svému zraku 5

Přestal jsem to dělat kvůli jiným důvodům nebo mne to nezajímá 6

11) Kvůli vašemu zraku, jak je pro vás obtížné vidět, jak lidé reagují na věci, které říkáte?

PŘEČTĚTE KATEGORIE POKUD BUDE TŘEBA a zakroužkujte jednu odpověď

Není to vůbec obtížné 1

Trochu obtížné 2

Středně obtížné 3

Extrémně obtížné 4

Přestal jsem to dělat kvůli svému zraku 5

Přestal jsem to dělat kvůli jiným důvodům nebo mne to nezajímá 6

12) Kvůli vašemu zraku, jak je pro vás obtížné vybrat si na sebe oblečení? (doma)

PŘEČTĚTE KATEGORIE POKUD BUDE TŘEBA a zakroužkujte jednu odpověď

Není to vůbec obtížné 1

Trochu obtížné 2

Středně obtížné 3

Extrémně obtížné 4

Přestal jsem to dělat kvůli svému zraku 5

Přestal jsem to dělat kvůli jiným důvodům nebo mne to nezajímá 6

13) Kvůli vašemu zraku, jak je pro vás obtížné setkávat se s lidmi u nich doma, na večírcích nebo v restauracích?

PŘEČTĚTE KATEGORIE POKUD BUDE TŘEBA a zakroužkujte jednu odpověď

Není to vůbec obtížné 1

Trochu obtížné 2

Středně obtížné 3

Extrémně obtížné 4

Přestal jsem to dělat kvůli svému zraku 5

Přestal jsem to dělat kvůli jiným důvodům nebo mne to nezajímá 6

14) Kvůli vašemu zraku, jak je pro vás obtížné jít do kina, divadla nebo na sportovní zápas?

PŘEČTĚTE KATEGORIE POKUD BUDE TŘEBA a zakroužkujte jednu odpověď

Není to vůbec obtížné 1

Trochu obtížné 2

Středně obtížné 3

Extrémně obtížné 4

Přestal jsem to dělat kvůli svému zraku 5

Přestal jsem to dělat kvůli jiným důvodům nebo mne to nezajímá 6

15) Nyní bych se rád zeptal/a na řízení auta. Řídíte, ať už pravidelně nebo alespoň občas?

Ano 1 pokračujte na 15c

Ne 2

15a) POKUD NE, ZEPTEJTE SE: Je to tak, že jste nikdy neřídil/a nebo řídil/a a přestal/a jste?

Nikdy neřídil 1 pokračujte na část 3, otázka 17

Přestal/a 2

15b) POKUD PŘESTAL/A: Bylo to hlavně kvůli vašemu zraku, kvůli jiným důvodům nebo kvůli vašemu zraku i jiným důvodům současně?

Hlavně kvůli zraku 1 pokračujte na část 3, otázka 17

Hlavně kvůli jiným důvodům 2 pokračujte na část 3, otázka 17

Zrak i jiné důvody 3 pokračujte na část 3, otázka 17

15c) POKUD V SOUČASNOSTI ŘÍDÍ: Jak je pro vás obtížné řízení během dne na místech, která znáte? Řekl/a byste, že

Není to vůbec obtížné 1

Trochu obtížné 2

Středně obtížné 3

Extrémně obtížné 4

16) Jak je pro vás obtížné řídit v noci? Řekl/a byste, že

Není to vůbec obtížné 1

Trochu obtížné 2

Středně obtížné 3

Extrémně obtížné 4

Přestal jsem to dělat kvůli svému zraku 5

Přestal jsem to dělat kvůli jiným důvodům nebo mne to nezajímá 6

16a) Jak je pro vás obtížné řízení za nepříznivých podmínek, jako je špatné počasí, dopravní špička, na dálnici nebo v městském provozu? Řekl/a byste, že

Není to vůbec obtížné 1

Trochu obtížné 2

Středně obtížné 3

Extrémně obtížné 4

Přestal jsem to dělat kvůli svému zraku 5

Přestal jsem to dělat kvůli jiným důvodům nebo mne to nezajímá 6

# Část 3 – reakce na problémy s viděním

Následující otázky se týkají toho, jak váš zrak ovlivňuje věci, které děláte. Pro každou otázku zkuste zodpovědět, zda je pro vás pravdivá neustále, většinou, občas, zřídkakdy nebo vůbec.

PŘEČTĚTE KATEGORIE a zakroužkujte jednu odpověď na každé řádce

|  |  | Neustále | Většinou | Občas | Zřídkakdy | Vůbec nikdy |
| --- | --- | --- | --- | --- | --- | --- |
| 17) | Dokážete méně, než byste chtěl/a, kvůli vašemu zraku? | 1 | 2 | 3 | 4 | 5 |
| 18) | Omezuje vás zhoršený zrak v tom, jak dlouho můžete pracovat nebo se věnovat různým činnostem? | 1 | 2 | 3 | 4 | 5 |
| 19) | Jak moc vám bolest či nepohodlí uvnitř nebo v okolí očí (např. pálení, svědění, bolest) brání dělat, co byste chtěl/a? | 1 | 2 | 3 | 4 | 5 |

Pro každý z následujících výroků, prosím řekněte, zda je pro vás určitě pravdivý, většinou pravdivý / většinou nepravdivý / určitě nepravdivý nebo si nejste jistí.

(zakroužkujte jednu odpověď na každé řádce)

|  |  | Určitě pravdivý | Většinou pravdivý | Nejsem si jistý/á | Většinou nepravdivý | Určitě nepravdivý |
| --- | --- | --- | --- | --- | --- | --- |
| 20) | Kvůli zhoršenému zraku většinu času zůstávám doma. | 1 | 2 | 3 | 4 | 5 |
| 21) | Kvůli zhoršenému zraku se často cítím frustrovaný/á. | 1 | 2 | 3 | 4 | 5 |
| 22) | Kvůli zhoršenému zraku mám nad tím, co dělám, mnohem menší kontrolu. | 1 | 2 | 3 | 4 | 5 |
| 23) | Kvůli zhoršenému zraku se musím příliš spoléhat na to, co mi řeknou ostatní lidé. | 1 | 2 | 3 | 4 | 5 |
| 24) | Kvůli zhoršenému zraku potřebuji hodně pomoci od ostatních. | 1 | 2 | 3 | 4 | 5 |
| 25) | Dělá mi starost, že kvůli zhoršenému zraku udělám něco, čím znemožním sebe nebo ostatní. | 1 | 2 | 3 | 4 | 5 |

TO JE KONEC ROZHOVORU. DĚKUJI VÁM ZA VÁŠ ČAS A POMOC.
