# Supplementary material for: The intraocular implant and visual rehabilitation improve the quality of life of elderly patients with geographic atrophy secondary to age-related macular degeneration
Source: Graefes Arch Clin Exp Ophthalmol. 2022 Aug 19;261(1):263–72. doi: 10.1007/s00417-022-05803-6 (PMC9388354; doi:10.1007/s00417-022-05803-6)
Supplement: Supplementary file 3 — Supplementary file3 (DOC 55 KB) [file 417_2022_5803_MOESM3_ESM.doc]

Name of the patient:

|  | **With operated eye …** *(insert the text on the line and ask how)****,* …** **than if I hadn't had the operation.** | Much worse | Worse a little | The same | Better a little | Much better | I cannnot assess |
| --- | --- | --- | --- | --- | --- | --- | --- |
| 1 | I can do housework (cleaning, cooking, minor repairs, etc.) | -- | - | 0 | + | ++ | / |
| 2 | I manage shopping | -- | - | 0 | + | ++ | / |
| 3 | My family life and relationships with loved ones are | -- | - | 0 | + | ++ | / |
| 4 | My social life and ability to make contacts with other people is | -- | - | 0 | + | ++ | / |
| 5 | I can take care of my appearance | -- | - | 0 | + | ++ | / |
| 6 | I feel physically | -- | - | 0 | + | ++ | / |
| 7 | I can move outside (on foot, by car, by train) | -- | - | 0 | + | ++ | / |
| 8 | I enjoy my hobbies and interests | -- | - | 0 | + | ++ | / |
| 9 | My self-confidence is | -- | - | 0 | + | ++ | / |
| 10 | My zest of life is | -- | - | 0 | + | ++ | / |
| 11 | I can do things on my own | -- | - | 0 | + | ++ | / |
| 12 | People react to me | -- | - | 0 | + | ++ | / |
| 13 | I'm losing personal belongings | -- | - | 0 | + | ++ | / |
| 14 | I like food and drink | -- | - | 0 | + | ++ | / |
| 15 | it takes me a long time to handle normal activities (dressing, buying, food, etc.) | -- | - | 0 | + | ++ | / |
| 16 | I have the pleasure of nature | -- | - | 0 | + | ++ | / |
| 17 | I can read | -- | - | 0 | + | ++ | / |
| 18 | I can write (messages, letters, etc.) | -- | - | 0 | + | ++ | / |
| 19 | I can watch TV | -- | - | 0 | + | ++ | / |
| 20 | The operation affected your life in some way that the questionnaire did not ask? | | | | | | |

*NOTE: Answer options: better-worse (questions 1, 2, 3,4, 5, 6, 7, 11, 12, 17, 18,19),
higher-lower (questions 9, 10),more-less (qutestions 8, 14),less – more (question 13), shorter time –longer (question 15), more – less (question 16). The first is always the positive option.*

Date: Name of the specialist:

NOTE 2: The English version of the questionnaire was created for the purposes of publication by translating the Czech version. During our study, we administered the Czech version only.
