# Supplementary material for: The intraocular implant and visual rehabilitation improve the quality of life of elderly patients with geographic atrophy secondary to age-related macular degeneration
Source: Graefes Arch Clin Exp Ophthalmol. 2022 Aug 19;261(1):263–72. doi: 10.1007/s00417-022-05803-6 (PMC9388354; doi:10.1007/s00417-022-05803-6)
Supplement: Supplementary file 4 — Supplementary file4 (DOC 53 KB) [file 417_2022_5803_MOESM4_ESM.doc]

Jméno dotazovaného:

|  | **S operovaným zrakem …** *(vložit text na řádku a doptat se jak)****,* … než kdybych operaci nepodstoupil/la.** | výrazně hůře | trochu hůře | stejně | trochu lépe | výrazně lépe | nelze posoudit |
| --- | --- | --- | --- | --- | --- | --- | --- |
| 1 | zvládám domácí práce (úklid, vaření, drobné opravy atd.) | -- | - | 0 | + | ++ | / |
| 2 | zvládám nákupy | -- | - | 0 | + | ++ | / |
| 3 | můj rodinný život a vztahy s nejbližšími jsou | -- | - | 0 | + | ++ | / |
| 4 | můj společenský život a schopnost navazovat kontakty s okolím je | -- | - | 0 | + | ++ | / |
| 5 | dokážu pečovat o svůj vzhled | -- | - | 0 | + | ++ | / |
| 6 | tělesně se cítím | -- | - | 0 | + | ++ | / |
| 7 | zvládám se pohybovat venku (pěšky, autem, vlakem) | -- | - | 0 | + | ++ | / |
| 8 | užívám si své koníčky a zájmy | -- | - | 0 | + | ++ | / |
| 9 | moje sebedůvěra je | -- | - | 0 | + | ++ | / |
| 10 | moje chuť do života je | -- | - | 0 | + | ++ | / |
| 11 | dokážu dělat věci samostatně | -- | - | 0 | + | ++ | / |
| 12 | na mě reagují lidé | -- | - | 0 | + | ++ | / |
| 13 | ztrácím osobní věci | -- | - | 0 | + | ++ | / |
| 14 | chutná mi jídlo a pití | -- | - | 0 | + | ++ | / |
| 15 | trvá mi zvládnout běžné činnosti (oblékání, nákup, jídlo atd.) | -- | - | 0 | + | ++ | / |
| 16 | mám potěšení z přírody | -- | - | 0 | + | ++ | / |
| 17 | dokážu číst | -- | - | 0 | + | ++ | / |
| 18 | dokážu psát (vzkazy, dopisy atd.) | -- | - | 0 | + | ++ | / |
| 19 | mohu sledovat televizi | -- | - | 0 | + | ++ | / |
| 20 | Ovlivnila operace váš život nějakým způsobem, na který se dotazník neptal? | | | | | | |

*POZN. Možnosti odpovědí: lépe – hůře (otázky 1, 2, 3, 5, 6, 7, 11, 12, 17, 18,19), lepší – horší (otázky 3, 4),
vyšší – nižší (otázky 9, 10), více – méně (otázka 8, 14), méně – více (otázka 13), kratší dobu – déle (otázka 15), větší – menší (otázka 16). První je vždy ta pozitivní varianta.*

Datum: Provedl:
